# Risk and prognosis of secondary bladder cancer after post-operative radiotherapy for gynecological cancer

**DOI:** 10.17305/bjbms.2021.6338

**Published:** 2021-10-29

**Authors:** Li Wen, Guansheng Zhong, Yingjiao Zhang, Miaochun Zhong

**Affiliations:** 1 Department of Prenatal Diagnosis and Screening Center, Hangzhou Women’s Hospital (Hangzhou Maternity and Child Health Care Hospital), Hangzhou, Zhejiang, China; 2 Department of Breast Surgery, The First Affiliated Hospital, College of Medicine, Zhejiang University, Hangzhou, Zhejiang, China; 3 Department of Gastroenterology, The 903 Hospital of the Joint Logistics Support Force of the Chinese People’s Liberation Army, Hangzhou, Zhejiang, China; 4 Department of General Surgery, Zhejiang Provincial People’s Hospital, Affiliated People’s Hospital, Hangzhou Medical College, Hangzhou, Zhejiang, China

**Keywords:** Gynecological cancer, secondary bladder cancer, radiation therapy, prognosis, SEER

## Abstract

The aim of this study was to investigate the impacts of radiation therapy (RT) on the occurrence risk of secondary bladder cancer (SBC) and on the patients’ survival outcome after being diagnosed with gynecological cancer (EC). The data were obtained from the SEER database between 1973 and 2015. Chi-squared test was used to compare the clinicopathological characteristics among the different groups. Fine and Gray’s competing risk model was used to assess the cumulative incidence and occurrence risk of SBC in GC survivors. Kaplan–Meier method was utilized for survival analysis. A total of 123,476 GC patients were included, among which 31,847 (25.8%) patients received RT while 91,629 (74.2%) patients did not. The cumulative incidence of SBC was 1.59% or 0.73% among patients who had received prior GC-specific RT or not, respectively. All EBRT (standardized incidence ratio (SIR) = 2.49, 95% CI [2.17-2.86]), brachytherapy (SIR =1.96, 95% CI [1.60-2.38]), and combinational RT modality groups (SIR =2.73, 95% CI [2.24-3.28]) had dramatically higher SBC incidence as compared to the US general population. Receiving EBRT (HR = 2.83, 95% CI [2.34–3.43]), brachytherapy (HR = 2.17, 95% CI [1.67–2.82]), and combinational RT modality (HR = 2.97, 95% CI [2.34-3.77]) were independent risk factors for SBC development. Survival detriment was observed in SBC patients who received RT after GC diagnosis, as compared to those who did not receive RT. In conclusion, patients who underwent RT after GC had an increased risk of developing bladder as a secondary primary cancer. A long-term surveillance for SBC occurrence is necessary for GC patients who have received prior RT.

## INTRODUCTION

Over the past 100 years, radiation therapy has been successfully used as adjuvant treatment modality for the management of gynecological malignancies thought to be at high risk of recurrence [1-3]. By depositing high physical energy of radiation on pelvic lymph node regions, post-operative radiation therapy (RT) can effectively treat possible micrometastasis disease and thus reduce tumor recurrence. It is anticipated that radiotherapy will continue to be an integral component in the treatment of endometrial, cervical, vaginal, vulvar, as well as some selected epithelial ovarian cancers [4]. Nevertheless, radiotherapy is also deemed as a double-edged sword. High doses of ionizing radiation can either directly or indirectly (by producing free radicals) damage the genome of the cell, resulting in acute and late toxicity [5]. One of the most serious late side effects is the increased risk of occurring a radiation-induced second primary malignancy [6-8].

Several studies report that RT history may contribute to the development of various secondary primary malignancies [9,10]. A previous study made by Gonzales et al. revealed that approximately 8% of the secondary solid cancers could be associated with RT [11]. Nevertheless, Wiltink et al. reported that no increased risk of secondary cancer after RT was observed in their meta-analysis consisting of >2500 pelvic cancers patients from randomized TME [12], PPRTEC-1 [3], and PORTEC-2 [13] trials [14]. Moreover, a decreased risk for developing prostate cancer after pelvic RT for rectal cancer was also reported in a previously published study [15]. Hence, whether the risk of developing secondary primary malignancy increases after RT remains controversial.

Specifically, the bladder is within the irradiation field when RT is conducted for gynecological cancer (GC) which is mainly located in the pelvic cavity. Considering the early and late toxicities associated with RT, the objective of the current study was to evaluate the impact of RT on the risk of SBC development in GC survivors and the prognosis of GC patients who suffered with SBC, using the SEER database. Our findings may provide an important clue for future RT selection, patient counseling, and development of prevention strategies among GC survivors who are at an increased risk for developing SBC.

## MATERIALS AND METHODS

### Database and case selection

We performed a retrospective cohort research using the custom Surveillance, Epidemiology and End Results (SEER) database [Incidence-SEER 9 Regs Custom Data (with additional treatment fields), November 2017 Sub (1973-2015)]. The SEER program, a database established by the National Cancer Institute of the U.S., collected data from cancer patients accounting for approximately 28% of the U.S. population [16]. The SEER*Stat software (version 8.3.8, National Cancer Institute, Washington, USA) was utilized to access the data from SEER database. With the permission from the SEER program office, patients who were diagnosed with gynecological cancer, including cervix uterus (site code C53.0-C53.9), corpus and uterus (site code C54.0-54.3, C54.8, C54.9, and C55.9), ovary (site code C56.9), and other female genital organs (site code C51.0-C51.9, C52.9, C57.0-C57.9, C58.9), were extracted from the SEER database between 1973 and 2015. Only GC patients who survived more than 5 years were eligible, because it is considered to take at least 5 years latency period from RT exposure to solid tumor occurrence [17]. Other exclusion criteria were as follows: (1) Patients younger than 18 years of age at diagnosis; (2) patients with unknown information for the race; (3) patients who did not undergo cancer specific surgery; (4) patients with unknown RT status; and (5) patients with gynecological cancer not as their first malignancy.

**FIGURE 1:**
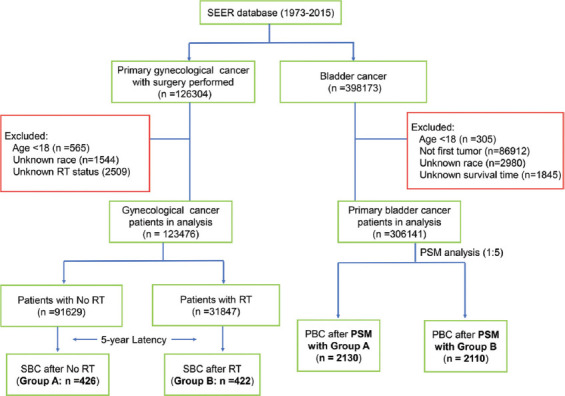
The flowchart of patients’ selection.

The eligible gynecological cancer patients were divided into two cohorts based on whether they received radiation therapy (RT) or not. Patients with subsequent SBC were eligible if they were diagnosed more than 60 months after gynecological cancer diagnosis. Patients in the no RT cohort who developed SBC were classified into Group A, while those in the RT cohort who developed SBC were classified into Group B. Moreover, patients who were diagnosed first with primary bladder cancer (PBC) between 1973 and 2015 were also included in the study. To reduce the possible selection bias in survival comparison, two cohorts of PBC patients were matched, respectively, for Groups A and B using the method of propensity score matching with a ratio of 5:1. The detailed flowchart for the patient’s selection is shown in Figure 1.

### Covariates and outcomes

Multiple variables were included in this study, including demographic characteristics (age, race, and years at diagnosis), disease characteristics (histological grade and stage), and treatment modalities (surgery, chemotherapy, and radiotherapy). Specifically, races, including American Indians, AK Natives, Asians, and Pacific Islanders, were classified into other races. The continuous variables, age or years at diagnosis, were transformed into categorical variables. According to the “radiation record” in the SEER database, radiotherapy for gynecological cancer was classified into external beam radiotherapy (EBRT), brachytherapy (radioactive implants), and combination of EBRT and brachytherapy. The primary outcome in this study was to evaluate the risk of SBC occurrence among patients who had or had not received GC-specific RT. The secondary outcome was to evaluate the impact of GC-specific RT on the overall survival (OS) and bladder cancer-specific survival (BCSS) among the SBC patients and compare it with the, respectively, matched PBC patients.

### Ethical statement

This study was based on public use deidentified data from the SEER database and did not involve interaction with human subjects or use personal identifying information. This study did not require informed consent from the SEER registered cases.

### Statistical analysis

Demographic and clinical characteristics between different cohorts were summarized by descriptive statistics and compared using the Pearson’s Chi-square test. The standardized incidence ratios (SIRs) for SBC after GC diagnosis were defined by calculating the ratio of observed-to-expected (O/E) incidence, which represented the change in the risk for developing SBC after GC diagnosis as compared to the general US population. The SIR analysis was performed using the SIR tools in the SEER program software (SEER*Stat 8.3.6). To evaluate the risk of developing SBC dynamically, the SIRs were stratified by latency time since GC diagnosis, age at GC diagnosis, and year of GC diagnosis.

The univariate and multivariable Fine and Gray competing risk regression model was utilized to evaluate the risk of developing SBC after GC diagnosis. Variables with *p* < 0.05 in univariable analyses were included in multivariable analyses. Specifically, SBC occurrence was considered as an event and all non-SBC caused deaths were defined as competing events. The cumulative incidence curve for SBC occurrence was plotted and compared by Gray’s test [18]. The Kaplan–Meier curves were plotted for the OS and BCSS between different cohorts, and the log rank test was used for the comparison of differences among the curves.

Descriptive statistics and Cox proportional hazards analysis were performed using the SPSS 24.0 (IBM Corp). The Fine and Gray competing risk analysis, cumulative incidence curve, and Kaplan–Meier curves were performed and plotted using the R software version 3.6.0. A two-sided *p* < 0.05 was considered statistically significant unless otherwise stated.

## RESULTS

### Patient characteristics

A total of 123,476 GC patients were finally extracted from the SEER database, among which 77,589 (62.8%) were uterine cancer, 18,676 (15.1%) were cervical cancer, 20,657 (16.7%) were ovarian cancer, and 6554 (5.3%) were other cancers. Among those GC patients, 31,847 (25.8%) patients had received RT, while 91,629 (74.2%) patients had not received RT. In comparison with patients who did not receive RT, patients who received RT were older, had an earlier diagnosis, had poorer histological differentiation, and mostly belonged to the White race and regional stage. A higher number of patients in the RT group had received chemotherapy as compared to the no RT group. Moreover, in the RT group, patients with younger age, lower histological grade, and at a later stage tended to receive EBRT or combinational RT modalities. In addition, patients with cervix cancer tended to receive combinational RT modalities while other types of GC patients were more likely to undergo EBRT. The detailed information for clinicopathological features among different groups are listed in Table 1. After 5-year latency since GC diagnosis, a total of 422 patients in the RT group and 426 patients in the no RT group were diagnosed with SBC at the end of the follow-up.

**TABLE 1:**
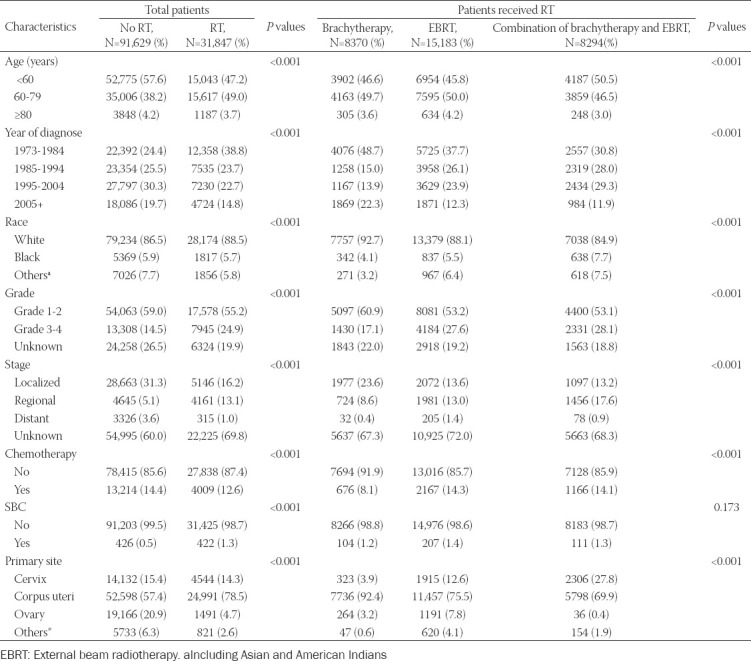
Baseline characteristics of patients with gynecological oncology (N=123,476)

### Cumulative incidence and SIR of SBC among GC survivors

**FIGURE 2:**
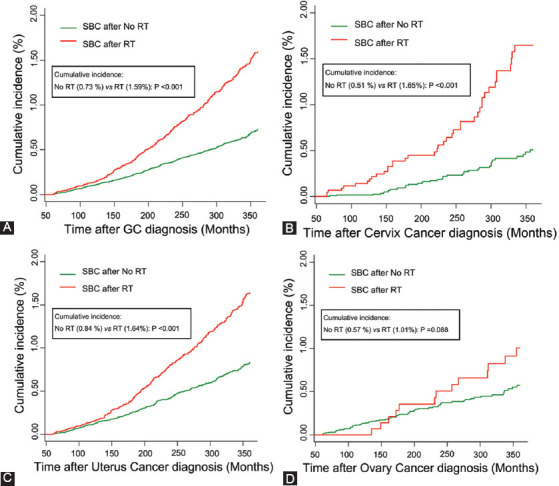
The cumulative incidence of secondary bladder cancer (SBC) in GC survivors. (A) Comparison of cumulative incidence between GC patients who received RT and those who did not receive RT; (B) comparison of cumulative incidence between cervix cancer patients who received RT and those who did not receive RT. (C) Comparison of cumulative incidence between uterus cancer patients who received RT and those who did not receive RT. (D) Comparison of cumulative incidence between ovary cancer patients who received RT and those who did not receive RT. P values were calculated with the Gray test. RT: Radiation therapy; SBC: Secondary bladder cancer; GC: Gynecological cancer.

**FIGURE S1:**
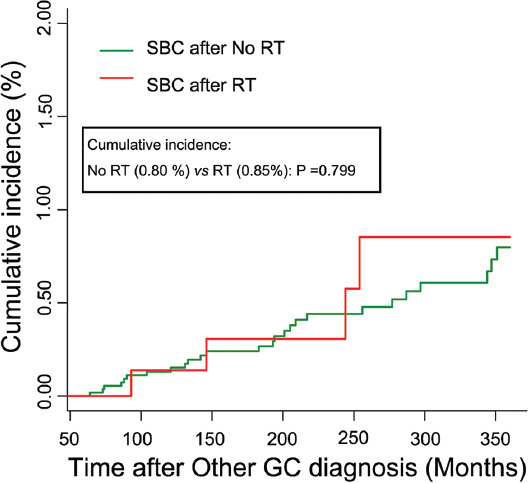
Cumulative incidence of patients with other types of gynecological cancer who received RT or not.

The cumulative incidence of SBC in GC patients who received prior RT or not was compared in this study. As shown in Figure 2A, GC patients who received RT were more likely to develop SBC than patients who had not received RT, with a cumulative incidence being 1.59% and 0.73% (*p* < 0.001), respectively, at the end of follow-up. In subgroup analysis, our data showed that the cumulative incidence of SBC between RT and no RT groups remained significant in patients with cervix (1.65% vs. 0.51%; *p* < 0.001) and uterus cancer (1.64% vs. 0.84%; *p* < 0.001), but not in ovary (1.01% vs. 0.57%; *p* = 0.088) and other types of GC (0.85% vs. 0.80%; *p* = 0.799) (Figure 2B-D and Figure S1).

The SIRs of SBC were also calculated for GC survivors in different RT modalities. Compared with the US general population, the incidence of SBC was dramatically high in all of the RT groups, including brachytherapy (SIR = 1.96, 95% CI [1.60–2.38]), EBRT (SIR = 2.49, 95% CI [2.17–2.86]), and combination of brachytherapy and EBRT (SIR = 2.73, 95% CI [2.24–3.28]) (Table 2). Nevertheless, a similar incidence risk of SBC was found in GC survivors who did not receive RT (SIR =1.06, 95% CI [0.96–1.16]). In subanalyses, SIR for SBC was stratified by latency time after GC diagnosis, year of GC diagnosis, age at GC diagnosis, and primary site of GC. As shown in Table 2, no significant change in incidence was observed among the patients who did not receive RT in all subgroups, when compared with the US general population. In latency SIR subanalyses, GC patients who had undergone prior RT had significantly higher SBC incidence, especially after more than 10 years of follow-up. Importantly, our data indicated that the incidence rate increased dramatically with the prolongation of follow-up time. For subanalyses of age or year of GC diagnosis, patients who were younger or diagnosed since 2005 had a dramatically increased risk of developing SBC. Moreover, a significantly elevated incidence of SBC was observed in almost all RT subgroups with different tumor site (Table 2).

**TABLE 2:**
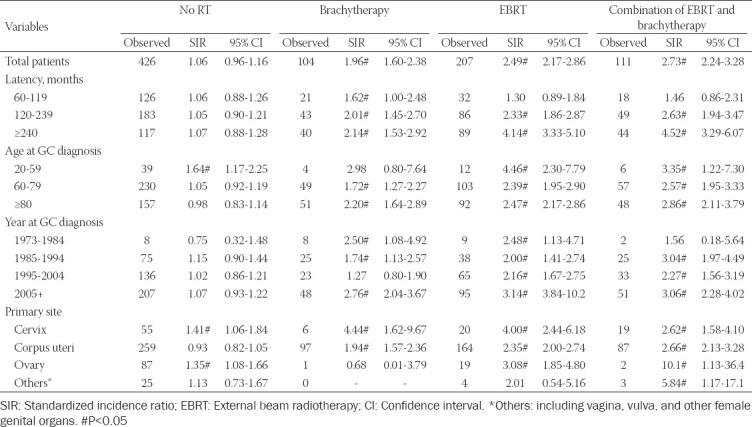
Standardized incidence ratio of secondary bladder cancer in patients with gynecological cancer

### Impact of RT on risk for developing SBC among GC survivors

To further investigate the effects of RT on SBC occurrence, univariate and multivariate competing risk regression analyses were conducted (Table 3). In multivariate analysis, we demonstrated that receiving GC-specific RT (HR = 2.69, 95% CI [2.29–3.16]) was significantly associated with an elevated risk for developing SBC. After stratifying the RT modality, the multivariate analysis further demonstrated that brachytherapy (HR = 2.17, 95% CI [1.67–2.82]), EBRT (HR = 2.83, 95% CI [2.34–3.43]), and combinational RT modality (HR = 2.97, 95% CI [2.34–3.77]) were independent risk factor for SBC occurrence among GC survivors.

**TABLE 3:**
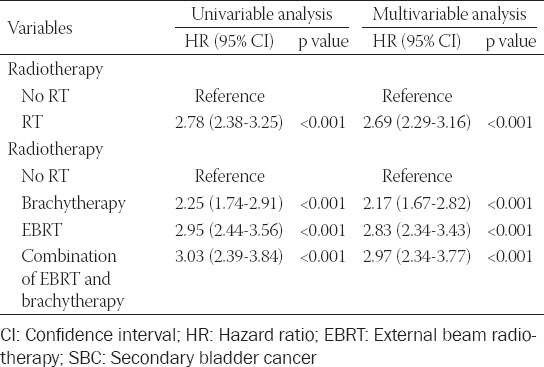
Univariable and multivariable competing risk analysis of risk of developing SBC in gynecological cancer survivors

### Impact of RT on survival of SBC among GC survivors

**FIGURE 3:**
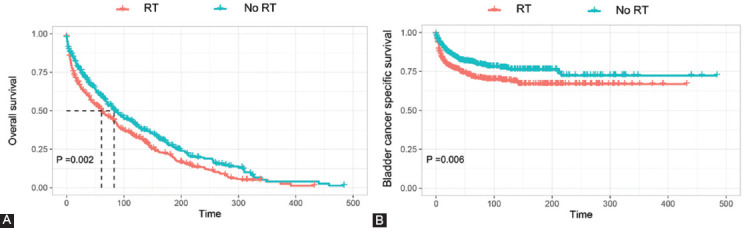
Kaplan–Meier curve of OS (A) and BCSS (B) in SBC patients who received RT or not after GC diagnosis. OS: Overall survival; BCSS: Bladder cancer-specific survival; RT: Radiation therapy; GC: Gynecological cancer.

**FIGURE 4:**
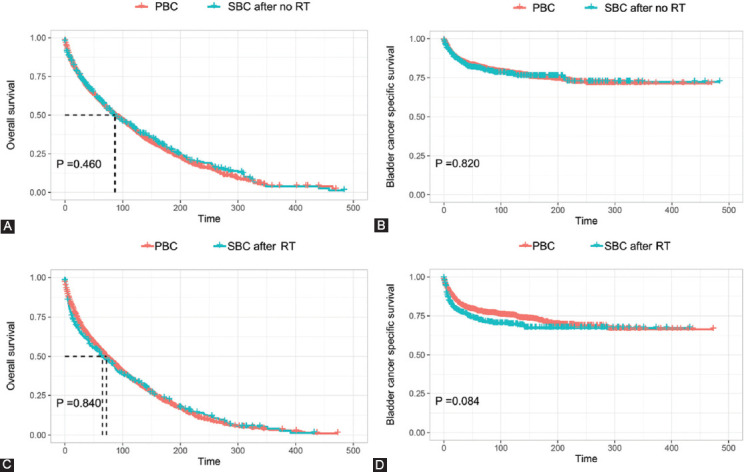
Comparison of survival between PBC patients and SBC patients who received RT or not after GC diagnosis. (A) OS between PBC and SBC after no RT; (B) BCSS between PBC and SBC after no RT; (C) OS between PBC and SBC after RT; (D) BCSS between PBC and SBC after RT. OS, Overall survival; BCSS: Bladder cancer-specific survival; RT: Radiation therapy; PBC: Primary bladder cancer; SBC: Secondary bladder cancer; GC: Gynecological cancer.

Both of the OS and BCSS were compared for SBC patients who underwent RT and those who did not in this study. As shown in Figure 3, the Kaplan–Meier curves showed that patients who had received prior RT had significantly inferior OS and BCSS as compared to those who had not received RT. Subsequently, using the PSM method, two cohorts of primary bladder cancer patients were matched separately for SBC patients who received RT or not after GC diagnosis. After adjusting for propensity scores, all features were well-balanced between matched PBC patients and SBC patients after GC diagnosis (Table S1-S2). As shown in Figure 4, two cohorts of matched PBC patients all had similar OS and BCSS in comparison with SBC patients regardless of receiving RT or not after GC diagnosis.

## DISCUSSION

The present study concentrated on evaluating the impact of receiving prior RT on the risk of developing SBC among GC survivors and on the prognosis of subsequent SBC. The current data showed that the cumulative incidence of SBC among GC patients who underwent prior RT was significantly higher than patients who did not receive RT. All of the RT modalities were demonstrated as independent risk factors for developing SBC among GC survivors. A survival detriment was observed in SBC patients who received prior RT after GC diagnosis, when compared with those who did not receive RT.

Several previous studies have evaluated the risk for SBC development among patients who had received pelvic RT for their pelvic cancer, with varying results. A publication by Wiltink et al. pooled the data for a total of 2500 EC or RC patients from three randomized trials and found that patients who received brachytherapy or EBRT had no increased risk of occurrence of SBC compared with patients who received surgery alone [14]. In a randomized trial reported by Onsrud et al., 568 patients with Stage I endometrial cancer were randomly assigned to either vaginal radium brachytherapy (VBT) followed by EBRT or VBT alone. An increased risk (HR = 1.42, 95% CI [1.01-2.00]) of secondary cancer was observed in the EBRT group compared with the control group. Importantly, the proportion of SBC was higher in the EBRT group (3.7%) than in the control group (2.6%) [19]. However, the small sample size, with only 13 SBC observed, caused limited statistical power for the conclusion. Wang et al. assessed the risk of developing secondary cancer in rectal cancer (RC) survivors receiving pre- or post-operative RT using the Taiwan’s National Health Insurance Research Database [20]. Their result showed an increased risk of developing SBC for patients received post-operative RT but not for those received pre-operative RT.

When death is not considered as a competing event, the probability of developing SBC may be overestimated as several patients die before SBC occurrence. Hence, the Fine-Gray competing risk model was used in our study to analyze the risk of developing SBC. We found that GC patients who received RT had an increased incidence of SBC compared with those who did not receive RT. This could be attributed to the fact that the typical radiation fields of both EBRT and vaginal cuff brachytherapy for GC include a portion of the bladder. However, the subgroup analysis of ovary or other types of GC showed no significant difference for the cumulative incidence of SBC between RT and no RT group, which might be attributable to the limited number of RT cases. Although no statistical difference, a rapid increase in the cumulative incidence of SBC still could be observed at the later period of follow-up. Our results also indicated that EBRT could result in a higher risk of developing SBC than brachytherapy, which could be explained by the dose-dependent effect of RT. Indeed, the subgroup receiving a combination of EBRT and brachytherapy had the relatively highest incidence and risk of SBC occurrence. A similar dose-dependent association was reported about the second cancers after pelvic RT for cervical cancer [21,22].

In addition, the SIR analysis in our study showed a significantly high probability of developing a SBC among GC survivors who had received prior RT, as compared to the US general population. The result echoed previous studies concentrating on evaluating the risk of secondary primary cancer in EC survivors [23,24]. However, our data also confirmed that GC survivors who did not receive RT had a similar incidence risk for developing SBC as compared to the US general population, which further implied that SBC may be induced by RT treatment. We also found that the incidence of SBC increased with the prolongation of follow-up time after GC diagnosis, especially after a latency of over 10 years. At present, the primary objective of surveillance in GC survivors is to detect recurrence or metastasis within 3-5 years of follow-up [25]. However, our data would suggest that patients who received RT may benefit from long-term detection of SBC. Regarding the effect of age on the risk of SBC occurrence, our data showed that the younger GC survivors who received RT had a highest risk of developing SBC as compared to elderly patients. A possible explanation might be that a relatively longer life expectancy would increase the risk of SBC occurrence. Moreover, the SBC incidence increased, especially since 2005, but not in any RT group. This tendency might be due to the increasing number of GC survivors which is caused by advancement of RT technology.

The results from multivariate competing risk analysis and SIR analysis, taken together, indicated that patients who underwent RT after GC diagnosis had an increased risk for developing bladder as a secondary primary cancer. The highest incidence of SBC was found after a latency of over 20 years. It is of great clinical implication that a long-term surveillance for the detection of SBC is necessary for GC survivors after treatment of RT. In addition, it is also a very important clinical issue to investigate the impact of GC-specific RT on prognosis of subsequent SBC. Hence, survival analyses were performed to compare OS and BCSS of SBC after RT with those without receiving RT. Our data demonstrated that patients who received prior RT had significant inferior survival as compared to patients who did not receive RT. We suspect that SBC after RT might have different biological behavior due to induction of distinct tumorigenic signaling pathways after radiation exposure. By means of the PSM method, we also demonstrated no significant difference in survival between PBC and SBC with or without prior RT history. This result was in line with several previous studies which demonstrate that a prior cancer history has no effect on survival in other cancers [26-28].

Several limitations exist in our study. First, potential biases were inherent in our study due to the intrinsic weaknesses of retrospective databases. The occurrence of secondary bladder cancer may not only be associated with radiation exposure but may also be affected by other crucial risk factors which could not be completely balanced due to lack of information in SEER database, such as smoking history, the use of chemotherapeutic agents (cisplatin and cyclophosphamide), and even genetic background. Second, the effect of dose, fractionation, and timing of RT on the risk of SBC could not be determined, because such information was also unavailable in the SEER database. We believe that all the observed results in this study should be prospectively validated.

## CONCLUSION

The current study showed that patients who underwent RT for a primary gynecological cancer had an increased risk for developing bladder cancer as a secondary primary cancer. A prior GC-specific RT had an adverse impact on the survival of SBC patients. There were no significant survival differences between PBC and SBC patients with or without prior GC-specific RT history.

## Figures and Tables

**TABLE S1:**
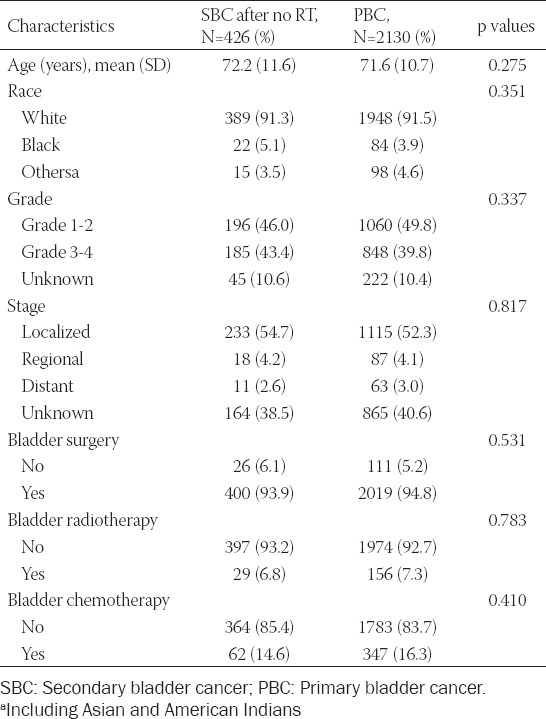
Baseline characteristics of SBC patients who received no prior GC specific RT and matched PBC patients

**TABLE S2:**
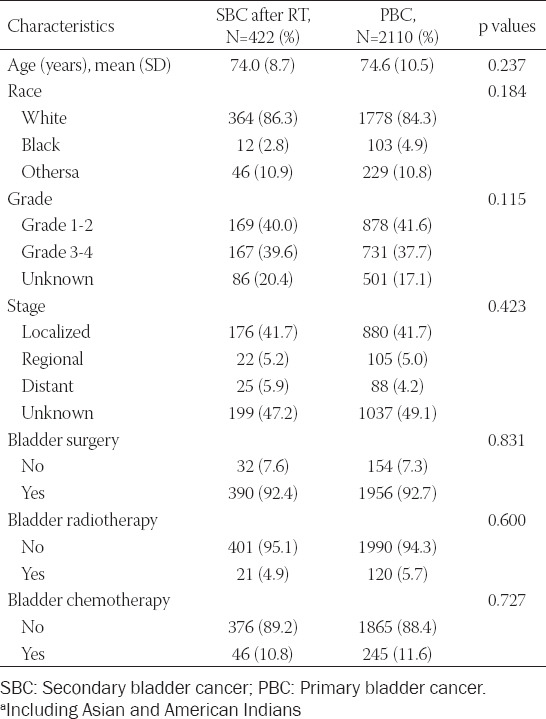
Baseline characteristics of SBC patients who received prior GC-specific RT and matched PBC patients
